# Support infrastructure available to Canadian residents completing post-graduate global health electives: current state and future directions

**Published:** 2016-12-05

**Authors:** Lojan Sivakumaran, Tasha Ayinde, Fadi Hamadini, Sarkis Meterissian, Tarek Razek, Robert Puckrin, Johanna Munoz, Shawna O’Hearn, Dan L Deckelbaum

**Affiliations:** 1 McGill University; 2 Global Health Programs, McGill University; 3 Centre for Global Surgery, McGill University Health Centre; 4 Dalhousie University, Global Health

## Abstract

**Background:**

Global health electives offer medical trainees the opportunity to broaden their clinical horizons. Canadian universities have been encouraged by regulatory bodies to offer institutional support to medical students going abroad; however, the extent to which such support is available to residents has not been extensively studied.

**Methods:**

We conducted a survey study of Canadian universities examining the institutional support available to post-graduate medical trainees before, during, and after global health electives.

**Results:**

Responses were received from 8 of 17 (47%) Canadian institutions. Results show that trainees are being sent to diverse locations around the world with more support than recommended by post-graduate regulatory bodies. However, we found that the content of the support infrastructure varies amongst universities and that certain components—pre-departure training, best practices, risk management, and post-return debriefing—could be more thoroughly addressed.

**Conclusion:**

Canadian universities are encouraged to continue to send their trainees on global health electives. To address the gaps in infrastructure reported in this study, the authors suggest the development of comprehensive standardized guidelines by post-graduate regulatory/advocacy bodies to better ensure patient and participant safety. We also encourage the centralization of infrastructure management to the universities’ global health departments to aid in resource management.

## Introduction

Post-graduate global health (GH) electives are valuable opportunities for residents to increase knowledge,^1^ diversify their skillset,^2^ and develop cultural sensitivity.^3^ In 2009, a review of studies showed that residents are increasingly incorporating the availability of GH electives into their selection criteria of residency programs.^4^ Residents who have completed GH electives have also been found to be more confident, have greater sensitivity to cost issues, rely less on technology, and have a better appreciation for cross-cultural communication.^5^

GH electives call residents to adapt to new challenges; this makes support infrastructure from the home and host institutions an important resource.^6^,^7^ Such infrastructure includes pre-departure training (PDT), bodily-fluid exposure protocols, best practice guidelines, and post-return debriefing (PRD). The Royal College of Physicians and Surgeons of Canada (RCPSC) provides a modest framework (see Appendix A) for the organization of GH electives.^8^ There are no such guidelines available from the College of Family Physicians Canada.

Given recent initiatives to standardize the GH elective infrastructure available to medical students - particularly in regards to PDT^9^,^10^ - as well as the publication of various guidelines for postgraduate GH electives,^4^,^11^–^15^ it is of interest as to what infrastructure is actually available to Canadian residents and whether there is a gap between what is offered and what could be considered ideal. The goal of the present study was to examine these issues using a survey of Canadian post-graduate programs.

## Methods

A survey created using Limesurvey™ was sent to the post-graduate deans of the 17 Canadian medical programs to determine the current state of Canadian GH elective infrastructure (see Appendix B). Questions were developed after a literature review with the help of GH leaders at McGill University. The survey was tested at the home institution before distribution. If the post-graduate dean could not answer the survey, responses were accepted from other global health authorities. Open text responses were allowed in case of perceived question ambiguity. Responses were collected from April 2014 to January 2015. Descriptive statistics were performed using Microsoft Excel™ by author LS. The McGill Institutional Research Board approved this project.

## Results

Twelve complete survey responses were received. These were completed by universities’ global health directors or post-graduate vice-deans; one respondent, belonging to a global health department, self-identified as “physician.” In certain cases, completed surveys were received from multiple individuals involved with global health at a single institution; these were synthesized to one response per university by including positive-over-negative responses (as one individual was likely aware of infrastructure that the other individual was not). In total, responses were received from eight of 17 universities (47%): McGill University, University of Ottawa, Laval University, University of Toronto, University of Alberta, University of British Columbia, Queen’s University, and one anonymous institution. Responses were randomly coded to maintain anonymity.

**Figure 1:**
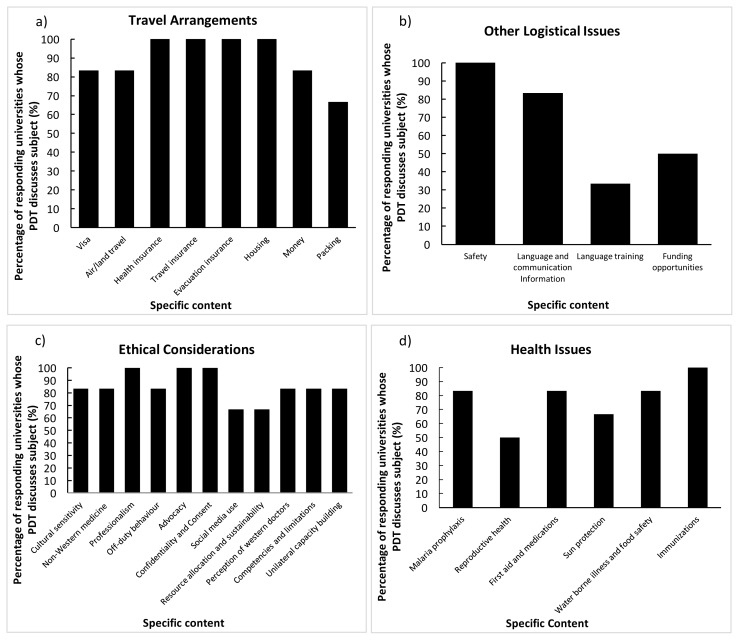
Percentage of responding universities that cover specified content during their pre-departure training of residents within the framework of a) Travel arrangements, b) Other logistical issues, c) Ethical considerations, and d) Health issues. (n=6)

Six of the eight responding universities offer PDT for residents pursuing GH electives. The training is mandatory at three of these institutions. Of the three universities with non-mandatory PDT, two are aiming to make the training mandatory; the remaining university has its residents sign a guideline document. The content covered during PDT by the six universities is outlined in Figure 1.

**Figure 2:**
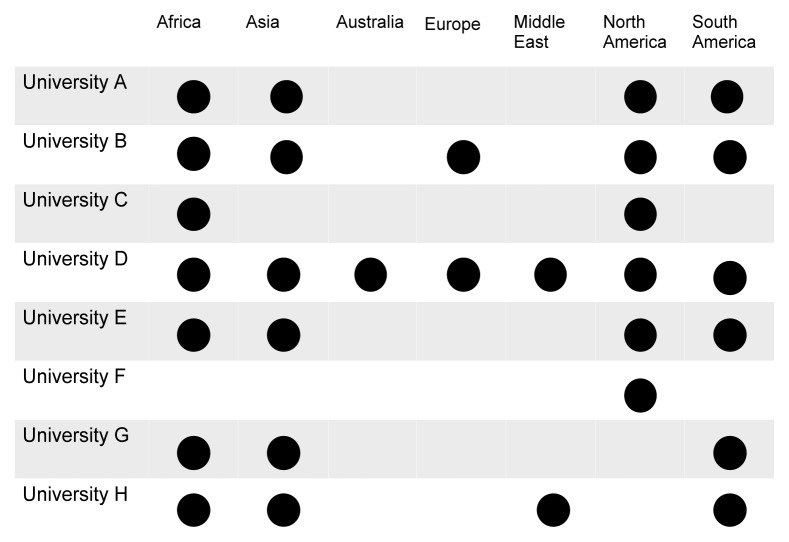
Locations to which responding Canadian universities send their post-graduate medical trainees for global health electives.

The locations to which universities send their post-graduate trainees are outlined in Figure 2. Three universities permit students to work in Department of Foreign Affairs and International Trade level two or three countries while five universities restrict travel to level one settings (where higher levels—from one to four—refer to higher risk areas). All universities have a supervisor at the host institution and all but one have a supervisor at the home institution. Two of the eight universities offer best practice guidelines for practicing medicine overseas; one of eight provides a formal code of ethics. Travel registries are available at five of eight institutions; mandatory enrolment is required in three, with one additional university transitioning to mandatory enrolment.

Regarding safety, five of the eight universities require participants to sign waivers outlining the risks of practicing abroad before departure. Bodily fluid exposure protocols are available at four of eight universities. The components offered include incident reports (n=2), home-site contact (n=4), on-site contact (n=0), post-exposure assessment procedure (n=3), HIV prophylaxis (n=1), HCV prophylaxis (n=0), written post-exposure protocol (n=3), on-site testing (n=1), communication with local site (n=1), communication with home centre (n=3), designated support person (n=1), and post-exposure counselling (n=4).

Following elective return, seven of eight institutions provide a formal review process of the elective. PRD (post-return debriefing) is provided at three of eight institutions; all three discuss the topics of in-field support, training quality, ethical issues, safety, communication, housing, and cultural acclimatization.

## Discussion

GH electives provide the opportunity for medical trainees in diverse settings to develop both their clinical and cultural competencies. The results of this survey are encouraging, suggesting that Canadian residents are completing electives across the globe with more infrastructure than currently mandated by bodies such as the RCPSC; however, there is still room for growth. The present study has identified four potential areas of development regarding Canadian GH elective infrastructure: PDT, professionalism, risk management, and PRD.

PDT is available at the majority of institutions with varied coverage of content. This availability likely traces its roots back to the push for mandatory PDT for Canadian medical students^9^,^10^ and to the growing support of PDT by resident advocacy bodies.^14^,^15^ While post-graduate PDT has yet to be standardized, many guidelines have been published to help institutions develop more evidence-informed training.^7^,^11^ More uniform adoption will be required to bridge the gap between undergraduate and postgraduate training.

Professional guidelines are another resource that could be made more consistently available. During GH electives, residents may be exposed to ethical dilemmas beyond the scope of their home training.^16^ Professional/ethical frameworks—in addition to the guidance of the host supervisor—would help trainees to better navigate these dilemmas. These standards could be developed at the level of the institution or at the level of the college and ideally should offer program-specific guidance.

Post-exposure resource availability is another important gap in GH elective infrastructure. Since medical trainees under-report bodily fluid exposure,^17^–^19^ the finding that only half of responding universities have post-exposure infrastructure—with inconsistent coverage of content—is concerning. The authors recommend the standardization of safety resources for GH electives, with consideration of the resources outlined in the Results section.

Finally, universities should be encouraged to uniformly offer PRD. PRD is a continuation of a process that begins with PDT that allows for the appraisal of the elective in the context of GH objectives;^7^,^20^ it is also helpful to address any moral distress that may have been incurred during the elective. Similarly to PDT, there is no standardized Canadian PRD. However, there are published guidelines that offer institutions a reasonable template for its development.^7^,^11^,^20^

Looking towards the future, it is necessary to address the barriers that prevent institutions from addressing gaps in their programming. Such barriers include the absence of guidelines by regulatory bodies, limited funding, lack of buy-in by residents and programs, and scheduling difficulties. To tackle these issues, the authors suggest recruiting organizational support on multiple fronts. Firstly, bodies that represent residents should consider educating trainees about the value of GH elective infrastructure and advocate for greater robustness. Secondly, medical colleges should consider developing standardized guidelines for residents performing GH electives, with specific regards to PDT, professionalism, risk management and PRD. Such a task has already been undertaken by certain colleges in the United States.^12^ Finally, to address scheduling and financial issues, each university should consider recruiting a centralized body—such as the global health department—to organize post-graduate elective infrastructure. While this may require initial financial investment at the onset, the pooling of resources among programs would likely decrease overall costs in comparison to establishing independent, overlapping infrastructure. These modifications will ideally help improve the safety profiles of GH electives and allow residents to provide more meaningful service across the globe.

### Limitations

One limitation of the present study was the response rate. We suspect that programs that do not offer GH electives elected not to respond to the survey. Regardless, the survey was considered geographically representative of Canadian medical institutions (with the exception of Atlantic Canada). A second limitation was the difficulty of assessing GH elective infrastructure by university. Since residency programs often organize GH infrastructure independently, the survey respondent may not have been fully aware of all the programming available at their university. Again, this points to the utility of a centralized GH body to disseminate information within, and eventually amongst, universities. In the meantime, future studies examining the availability of GH elective infrastructure by program would be useful to gauge the growth of elective infrastructure.

### Conclusions

Canadian medical programs are offering more institutional support to their trainees abroad than mandated; however, there is potential for further development. To address the gaps in GH elective infrastructure, the authors suggest the development of more comprehensive, standardized infrastructure by regulator/advocacy bodies. We also suggest the centralization of this infrastructure to GH departments to ensure widespread infrastructure availability amongst programs.

## Appendix A

Requirements for the organization of a global health elective as per the RCPSC (12)

1. The resident must be enrolled in a recognized program
2. The elective must be less than six months in duration
3. Planning must occur between the resident and program director; the program director must approve of the elective
4. There is a defined elective supervisor
5. There are defined educational objectives
6. There is an in-training evaluation system

## Appendix B

### Survey questions

1. Please state your title and university affiliation (Open Text Response)
2. Do you have post-graduate trainees participating in Global Health electives in the following regions?
  - Africa (Y/N)
  - Asia (Y/N)
  - Australia (Y/N)
  - Europe (Y/N)
  - Middle East (Y/N)
  - North America (Y/N)
  - South America (Y/N)
3. Do post-graduates trainees have a designated supervisor at the following locations?
  - At their home university/within their department? (Y/N)
  - At the host institution and/or organization? (Y/N)
  - Comment (Open Text Response)
4. Does your faculty offer pre-departure training to post-graduate trainees? (Y/N, Open Text Response)
5. Is the pre-departure training mandatory? (Y/N)
6. Does it include the following topics?
  - Respecting Travel Arrangements:
    i. Passport (Y/N)
    ii. Visa requirements (Y/N)
    iii. Air and Land Travel (Y/N)
    iv. Mandatory Health Insurance (Y/N)
    v. Travel Insurance (Y/N)
    vi. Evacuation Insurance (Y/N)
    vii. Housing (Y/N)
    viii. Money (Y/N)
    ix. Packing (Y/N)
  - Respecting health issues:
    x. Malaria prophylaxis (Y/N)
    xi. Reproductive health (Y/N)
    xii. Other medications and First Aid preparedness (Y/N)
    xiii. Sun protection (Y/N)
    xiv. Water borne illness and Food Safety (Y/N)
  - Respecting other logistics issues:
    xv. Immunizations/Vaccinations (Y/N)
    xvi. Safety (Y/N)
    xvii. Language and communication (Y/N)
    xviii. Funding opportunities (Y/N)
  - Respecting ethical issues:
    xix. Cultural competency and sensitivity (Y/N)
    xx. Understanding of non-Western medical practices and standards (Y/N)
    xxi. Professionalism (Y/N)
    xxii. Off-duty behaviour (Y/N)
    xxiii. Advocacy (Y/N)
    xxiv. Confidentiality and Informed Consent (Y/N)
    xxv. Considerations for use of social media (Y/N)
    xxvi. Resource allocation and sustainability (Y/N)
    xxvii. Perception of Western medical practitioners (social biases) (Y/N)
    xxviii. Questions around scope of practice and appropriate competency levels (limitations) (Y/N)
    xxix. Issues around unilateral capacity building (Y/N)
7. Who administers the pre-departure training (which department/office)? (Open Text Response)
8. Please list any other pre-departure training offered by your faculty that were omitted from previous questions? (Open Text response)
9. Does your faculty provide post-graduate trainees with Best Practices Guidelines for practicing medicine oversees? (Y/N, Open Text Response)
10. Does your faculty have a publicly available Code of Ethics for practicing overseas? (Y/N, Open Text Response)
11. Does your faculty offer cultural and/or language preparation resources to post-graduate trainees? (Y/N, Open Text Response)
12. Does your faculty have a publicly available Code of Ethics for practicing overseas?
13. Do you have a travel registry for post-graduate trainees? (Y/N, Open Text Response)
  - If yes, is it mandatory? (Y/N, Open Text Response)
14. What is the safety standard applied for location of travel? Please select the highest DFAIT country level permitted.
  - DFAIT level 2 country allowed (Y/N)
  - DFAIT level 3 country allowed (Y/N)
  - DFAIT level 4 country allowed (Y/N)
  - Other: (Open Text Response)
15. Are post-graduate trainees required to sign a waiver, acknowledging the risks inherent in traveling and practicing medicine abroad? (Y/N, Open Text Response)
16. Is there a needle-stick injury or blood & bodily fluid exposure protocol in place? (Y/N, Open Text Response)
  - If yes, are the following included?
    - Incident report (Y/N)
    - Specific contact person on site (Y/N)
    - Contact person at the home university (Y/N)
    - Written post-exposure risk assessment procedure (Y/N)
    - Residents are required to travel with post-exposure prophylaxis for HCV (Y/N)
    - Residents required to travel with post-exposure prophylaxis for HIV (Y/N)
17. In the event of exposure, is there a protocol for further management? (Y/N)
  - If yes, are the following included? (Y/N)
    - On-site testing (Y/N)
    - Communication with local institution (Y/N)
    - Communication with home institution (Y/N)
    - Designated person for support (Y/N)
18. Does your faculty offer post-exposure counseling services? (Y/N)
19. Is there a formal reviewing process of electives? (Y/N)
20. Does your faculty offer formal post-placement debriefing? (Y/N)
  - If so, are the following addressed:
    - In-field support (Y/N)
    - Training quality (Y/N)
    - Ethical issues (Y/N)
    - Safety (Y/N)
    - Language (Y/N)
    - Housing (Y/N)
    - Cultural acclimatization (Y/N)
    - Other (Open Text Response)
